# Highly bioavailable Berberine formulation improves Glucocorticoid Receptor-mediated Insulin Resistance *via* reduction in association of the Glucocorticoid Receptor with phosphatidylinositol-3-kinase

**DOI:** 10.7150/ijbs.39508

**Published:** 2020-07-19

**Authors:** Zhaojie Meng, Yang Yu, Yining Zhang, Xuehan Yang, Xiaoyan Lv, Fengying Guan, Grant M. Hatch, Ming Zhang, Li Chen

**Affiliations:** 1 Department of Pharmacology, College of Basic Medical Sciences, Jilin University, Changchun, Jilin, China.; 2 Division of Biomedical Sciences, School of Medicine, University of California, Riverside, CA, United States of American.; 3 Key Laboratory of Medical Cell Biology, Institute of Translational Medicine, China Medical University, Shenyang, Liaoning Province, China.; 4 The First Hospital, Jilin University, Changchun, China.; 5 The Second Hospital, Jilin University, Changchun, China.; 6 Department of Pharmacology and Therapeutics, Center for Research and Treatment of Atherosclerosis, DREAM Manitoba Institute of Child Health, University of Manitoba, Winnipeg, Manitoba, Canada.

**Keywords:** Highly bioavailable berberine formulation, Huang-Gui solid dispersion, insulin resistance, phosphatidylinositol 3-kinase, glucocorticoid receptor

## Abstract

Excess glucocorticoid (GC) production is known to induce obesity and insulin resistance through increased activation of the glucocorticoid receptor (GR). The molecular mechanism for the non-genomic effects of excessive circulating GC on the insulin-signalling pathway in skeletal muscle is unknown. The plant alkaloid berberine has been shown to attenuate insulin resistance and inhibit gluconeogenesis in type 2 diabetic animals. A highly bioavailable berberine formulation termed Huang-Gui solid dispersion (HGSD), is a preparation of berberine coupled to sodium caprate and this markedly improving berberines bioavailability. Here we examined how HGSD treatment attenuated GR-mediated alteration in PI3K signalling and insulin resistance in diabetic rats, dexamethasone-treated mice and in insulin resistant C2C12 skeletal muscle cells. Blood glucose and skeletal muscle GC levels were increased and insulin signalling impaired in skeletal muscle of type 2 diabetic rats compared to controls. Treatment of these animals with HGSD restored blood glucose and skeletal muscle GC levels to that of controls. Insulin resistant C2C12 skeletal muscle cells exhibited impaired insulin signalling compared to controls and treatment of HGSD and RU486, an antagonist of GR, restored insulin signalling to that of control cells. Administration of dexamethasone to mice increased GR/GRα-associated PI3K and reduced IRS1-associated PI3K, phosphorylated-AKT, and membrane GLUT4 translocation resulting in a higher blood glucose concentration compared to controls. HGSD treatment of these mice improved insulin resistance by reducing the association of GR/GRα with PI3K. Excess GC-induced insulin resistance is mediated by increased association of GR with PI3K and treatment with HGSD attenuates these effects. We hypothesize that HGSD may be a promising candidate drug for the treatment of type 2 diabetes by reducing the association of GR with PI3K in skeletal muscle.

## Introduction

Excess glucocorticoid (GC) production (Cushing's syndrome) induces obesity and insulin resistance via activation of the intracellular glucocorticoid receptor (GR) 1, 2. Moreover, intracellular GR signalling determines tissue sensitivity to GCs and altered GR signalling is implicated in the development of type 2 diabetes and obesity. Pharmacological blockade of the GR in rats was shown to attenuate high-fat diet-induced adiposity, glucose intolerance, and insulin resistance 3. The GR is a cytoplasmic nuclear hormone receptor, which acts as a steroid ligand-activated transcription factor 4, 5. In rodents, increased hepatic *GR* mRNA induces activation of the key hepatic gluconeogenic enzyme phosphoenolpyruvate carboxykinase (PEPCK) leading to hyperglycaemia and insulin resistance in diabetic db/db mice and in obese Zucker rats 6-9. Skeletal muscle is a major target tissue for insulin-mediated glucose uptake, metabolism and utilization in humans. Treatment of C2C12 cells with retinoic acid attenuated the GC-mediated development of insulin resistance and impaired glucose tolerance 10. In human skeletal muscle cells, increased GR expression was assocaited with development of the metabolic syndrome 11, 12. To date, few studies have focused on the mechanism of how activation of the GR influences the insulin signalling pathway in skeletal muscle during the diabetic process.

Berberine (BBR) is a natural plant alkaloid isolated from the Chinese herb *Rhizoma coptidis* and is commonly used for the treatment of diarrhoea. Many studies have shown that BBR exhibits hypoglycaemic properties and ameliorates insulin resistance. We previously showed that BBR inhibited hepatic gluconeogenesis by decreasing the expression of PEPCK via activating 5'-adenosine monophosphate-activated protein kinase (AMPK) 13. In addition, BBR was shown to reduce dexamethasone (DEX)-mediated insulin resistance in theca cells *in vitro* 14. Therefore, BBR may regulate insulin signalling pathway in skeletal muscle via GC/GR pathway.

The low bioavailability and non-definitive mechanism of BBR action limit its clinical application as an antidiabetic drug. We previously showed that Huang-Gui solid dispersion (HGSD), a ternary drug delivery system with BBR, sodium caprate (SC, an intestinal absorption enhancer) and PEG6000, prepared by solid dispersion technology, dramatically enhanced a 4-fold increase in *in situ* intestinal perfusion and a 5-fold increase *in vivo* bioavailability of BBR 15, 16. Berberine has been confirmed to be the main active ingredient in HGSD, with no obvious alteration after SC treatment 17. HGSD treatment of high-fat diet and STZ-induced diabetic rats resulted in a markedly improved hypoglycaemic effect compared to BBR alone 15. In the present study, we utilized three distinct models of insulin resistance (high-fat diet, HFD) fed streptozotocin (STZ)-treated rats, insulin-resistant C2C12 cells, and dexamethasone (DEX)-treated mice to examine whether insulin resistance is mediated by the association of GR with PI3K and whether HGSD-treatment attenuates insulin resistance in these models.

## Materials and Methods

### Chemical Reagents

BBR (purity quotient >99.8%) was obtained from Northeast Pharmaceutical Group CO., LTD (Shenyang, China). RU486 and sodium caprate (SC) was purchased from Sigma-Aldrich CO. LLC (St Louis, MO, USA). PEG6000 was purchased from Tianjin Guangfu Fine Chemical Research Institute (Tianjin, China). Insulin and streptozotocin (STZ) were purchased from Sigma-Aldrich CO. LLC (St Louis, MO, USA). Glucose oxidase kit was purchased from BHKT Clinical Reagent CO., LTD (Beijing, China). Iodine [^125^I] insulin radioimmunoassay kit was purchased from Tianjin Nine Tripods Medical & Bioengineering CO., LTD (Tianjin, China). Dulbecco's Modified Eagle's Medium (DMEM), fetal bovine serum (FBS), penicillin/streptomycin and 0.25% trypsin EDTA solution were purchased from Gibco BRL (Grand Island, NY, USA). Polyclonal antibodies to IRS1, phosphorylated IRS1, AKT, phosphorylated AKT, PI3K, GLUT4, cMyc, MHC, GR, GRα and goat anti-rabbit IgG horseradish peroxidase conjugate were all purchased from Santa Cruz Biotechnology (Santa Cruz, CA, USA). GAPDH was purchased from Epitomics (Burlingame, CA, USA). ECL Western blotting Substrate was purchased from Pierce (Thermo Fisher Scientific, Rockford, IL, USA). Chemical reagents for Western blot analysis were obtained from Sigma, and polyvinylidene difluoride membranes were from Bio-Rad (Hercules, CA, USA). Other reagents were purchased from Beijing General Chemical Reagent Factory (Beijing, China).

### Animal experiments

Male Wistar rats (200-220 g) and ICR mice (18-22 g) were obtained from the Experimental Animal Holding Facility of Jilin University (certificate number: scxk2013-0003). All animals were housed in standard polypropylene cages (three rats or mice per cage) and maintained in an environmentally controlled breeding room (temperature: 20±2 °C, humidity: 60±5%, 12 h light/dark cycle). Animals were acclimated for at least 5 days and then processed for various experiments.

### Preparation of Huang-Gui solid dispersion

HGSD was prepared with BBR (the active ingredient), SC and PEG6000 following the weight ratio of 1:1:6 by solvent evaporation as previously described 15.

### *In vivo* animal models and drug administration

Rats were made diabetic as previously described 18. Control animals were fed regular rodent chow consisting of 5% fat, 53% carbohydrates, and 23% protein with a total calorific value of 25 kJ/kg. Rats were made diabetic by feeding a high-fat diet consisting of 22% fat, 48% carbohydrates, and 20% protein with a total calorific value of 44.3 kJ/kg for 4 weeks followed by intraperitoneal (i.p.) injection with 30 mg/kg streptozotocin (STZ) dissolved in 0.1 M sodium citrate buffer, pH 4.4, in volume of 2.5 ml/kg. Control rats received citrate buffer alone. After one week, fasting blood glucose (FBG) was measured using the glucose oxidase kit. Rats with fasting blood glucose (FBG) below 7.8 mmol/L were reinjected with STZ (30 mg/kg). After 4 weeks rats with fasting blood glucose above 7.8 mmol/L when measured twice were considered diabetic. Rats were then divided into 5 groups: (1) Control (treated with 0.5% sodium carboxymethyl cellulose, CMC-Na), (2) Diabetic (treated with 0.5% CMC-Na), (3) BBR treatment group (diabetic rats treated with 100 mg/kg BBR), (4) low-dose HGSD treatment group (diabetic rats treated with HGSD at an equivalent dose of 25 mg/kg BBR), (5) high-dose HGSD treatment group (diabetic rats treated with HGSD at an equivalent dose of 100 mg/kg BBR). All drugs were dissolved in preheated saline and administered via oral gavage daily for four weeks. All rats were allowed to continue to feed on their respective diets until the end of the study.

The insulin resistance mouse model was established by injection of DEX (i.p.) at a dose of 2 mg/kg for 7 consecutive days as previously described 19, 20. Control group mice were injected (i.p.) with saline in an equal volume of 5 mL/kg. The mice were divided into 4 groups and treated with the respective drugs via intragastric (i.g.) administration: (1) Control group (control mice receiving saline), (2) Model group (mice of the insulin resistance model receiving a matched volume of saline), (3) BBR treatment group (insulin resistant mice from the model group receiving 150 mg/kg BBR), (4) HGSD treatment group (insulin resistant mice from the model group receiving HGSD at an equivalent dose of 150 mg/kg BBR). Mice were then sacrificed after 7 days.

Intraperitoneal glucose tolerance test (IPGTT) or oral glucose tolerance test (OGTT) was conducted at the end of drug administration in both *in vivo* models. After a 12 h fast, glucose (2 g/kg) was administered (i.p. in rats and i.g. in mice). The blood was collected from the caudal vein at 0, 30, 60, 90 and 120 min after the glucose administration, and the glucose concentration in the serum samples was determined using the glucose oxidase kit.

Tissue was collected from animals after fasting for 12 h at the end point of each study. Rats were anaesthetized with 20% urethane (100 mg/kg), and blood samples were obtained from the abdominal aorta. The blood samples from mice were collected from the fundus vein. Blood samples were allowed to clot for 30 min at 4 °C and centrifuged (3,500 ×g, 10 min, 4 °C). The supernatant was used for measurement of glucose and insulin. The blood glucose was measured using the glucose oxidase kit. Insulin was measured by a radioimmunoassay. The skeletal muscle of animals was then collected after perfusion and sacrifice for protein evaluation. The levels of specific protein were determined by Western blot analysis.

### Preparation of HGSD conditioned serum

HGSD conditioned serum was prepared as previously described 21. Briefly, 18 rats were randomly divided into three groups and administered with gavages of HGSD, vehicle alone or saline. Rats were fed either 3 mL saline twice daily for 3 days or HGSD twice daily for 3 days at an equivalent dose of 1000 mg/kg BBR, which was estimated as 10 times the dose given to the diabetic rats from our previous study 22. 30 min after administration on the third day, blood was obtained from the aorta of the rats under sterile conditions and was allowed to coagulate at 25 °C for 4 h. The serum was separated by centrifugation at 3,000 rpm for 20 min. After filtration through a 0.45 μm cellulose acetate membrane twice, the serum was incubated in a 56 °C water bath for 30 min and then stored at -80 °C until use. The concentration of BBR in the HGSD conditioned serum detected by HPLC was 28.46±3.77 μM.

### C2C12 cell culture, differentiation and identification

C2C12 myoblast cells were maintained at subconfluent conditions in growth media containing DMEM with 4.5 g/L glucose, 100 U/mL penicillin, 100 μg/mL streptomycin, and 10% foetal bovine serum (Gibco BRL, Grand Island, NY, USA). All the cells were grown in a humidified 37 °C incubator with ambient oxygen and 5% CO_2_.

C2C12 cells at ~80% confluence were differentiated by incubation with 2% horse serum (HyClone, Logan City, Utah, USA). Cells were maintained for 3-7 days to obtain myotubes. After a 5 days myotubes were incubated with antibody (1:200) to myosin heavy chain (MF20, Santa Cruz, USA) and nuclei stained with Hoechst 33342 (1 µg/mL). Images were obtained using an immunofluorescence microscope.

### Cell viability

The cell viability was determined by MTT assay in 96 well tissue culture plates as described previously 23. After treatment, the culture medium was removed from the wells, and 200 μL of MTT reagent (Sigma) at a concentration of 1 mg/mL in PBS was added to each well. After 4 h incubation at 37 °C, the MTT reagent in PBS was removed, and the blue-coloured formazan product was solubilized in 0.15 mL of DMSO for 20 min. The absorbance of the converted dye was measured at a wavelength of 570 nm using a spectrophotometer.

### C2C12 cell insulin resistance model and drug treatment

After differentiation for 2 days C2Cl2 cells were incubated in the absence or presence of 100 nM insulin for 72 h to induce insulin-resistance. Cells were then randomly divided into groups: (1) Control non-insulin resistant cells treated +/- insulin, (2) insulin-resistant cells treated +/- insulin, (3) insulin-resistant cells treated +/- insulin and treated with 5 µM BBR, (4) insulin-resistant cells treated +/- insulin and treated with 5 μM HGSD conditioned serum, (5) insulin-resistant cells treated +/- insulin and treated with control vehicle serum, (6) insulin-resistant cells treated +/- insulin and treated with blank serum alone. In some experiments, insulin-resistant cells treated +/- insulin were treated with 1 μM RU486. All drugs were added 12 h before the end of the experiment.

### Glucose consumption and glucose uptake

The glucose consumption was measured as previously described 24, 25. The above cells were incubated in serum-free low-glucose (5.5 mM) DMEM overnight. Cells were then treated with insulin (100 nM) and the drugs in fresh serum-free low-glucose (5.5 mM) DMEM. After 12 h incubation, the medium was removed and glucose concentration in the medium was measured using the glucose oxidase kit. In some experiments, cells from each group were incubated with insulin (100 nM) 30 min before the end of the experiment.

After differentiation, the above cells were washed 3 times with KRB containing 0.5% BSA at 40 min intervals over 2 h at 37 °C and then preincubated with drugs and insulin (100 nM) for 3 h. Subsequently, cells were incubated in 200 µM 2-NBD-glucose in PBS for an additional 30 min and then washed 3 times with ice-cold PBS. The fluorescence intensity of the cells was determined using a fluorescence microplate. In some experiments, cells from each group were incubated with insulin (100 nM) for 10 min.

### GLUT4 translocation

The GLUT4 cell surface density was measured in fixed and non-permeabilized C2C12 myotubes using an antibody-coupled absorbance assay as described previously 26. Briefly, after being treated as described above, cells were serum-depleted for 2 h and then incubated plus or minus insulin (100 nM) for 10 min. The cells were then washed twice with ice cold PBS, fixed with 3% (v/v) paraformaldehyde at 4 °C for 10 min and then incubated at room temperature for an additional 20 min. All subsequent steps were performed at room temperature. Cells were then incubated with 0.1 M glycine in PBS for 10 min and then blocked with 5% skim milk (w/v) in PBS for 10 min. Incubation with anti-GLUT4myc polyclonal antibody (1:250) in 5% milk in PBS for 1 h was followed by five washes with PBS and incubation with HRP-conjugated goat anti-rabbit IgG (1:2000) for 1 h. The cells were extensively washed with PBS and incubated with 1 ml/well of 0.4 mg/mL o-phenylenediamine dihydrochloride reagent for up to 20 min. The reaction was stopped by the addition of 0.25 mL of 3 N HCL. The supernatant was collected, and the optical absorbance was measured at 492 nm. The background absorbance obtained from wild-type C2C12 myotube wells was subtracted from all other values.

### Western blot analysis

Skeletal muscle tissue (50 mg) and C2C12 cells were homogenized at 4 °C in 1 mL or 500 μL, respectively, of ice cold TES buffer (20 mM Tris-HCl, pH 7.4, containing 250 mM sucrose, 1 mM EDTA, 1 mM phenylmethylsulfonylfluoride, 0.01 mM leupeptin, and 5 μg/mL aprotinin). The lysate was centrifuged at 10,000 rpm for 10 min at 4 °C. Aliquots of the supernatant were removed for protein analysis by the Bradford method (Bio-Rad). The samples were denatured by boiling for 5 min and separated by 10% SDS polyacrylamide gel electrophoresis and then electroblotted onto polyvinylidene difluoride membranes (Bio-Rad) at 4 °C. After blocking with 5% (w/v) skim milk for 2 h at room temperature, the membranes were then incubated with respective primary antibodies with gentle agitation overnight at 4 ºC. The membranes were washed 3 times for 10 min each with 15 mL of TBST (10 mM Tris-HCl, 150 mM NaCl and 0.1% (v/v) Tween-20) and then incubated with secondary antibody (1:2000 horseradish peroxidase conjugated goat anti-rabbit or mouse IgG) at room temperature for 2 h. Protein bands were visualized with ECL on an X-ray film and then scanned and quantified using the Quantity One image analysis software (Bio-Rad).

### Skeletal muscle-specific corticosterone measurement

The 50 mg of skeletal muscle tissue was homogenized and then sonicated in 500 μL cold PBS. After centrifuging at 5,000 × g for 10 min, an aliquot of the supernatant was removed to determine corticosterone levels using the Corticosterone Enzyme Immunoassay kit (Catalog No. KGE009, R & D System) according to the manufacturer's instructions.

### Co-immunoprecipitation experiments

50 mg of skeletal muscle tissue was homogenized for 5 min in cold 1% Nonidet-P40. The mixture was kept on ice for 60 min before centrifugation at 10,000 × g for 10 min at 4 ºC. The supernatant (2 mg protein aliquots) was incubated with 4 μg anti-PI3K antibody overnight at 4 °C. Protein A/G (25 μL) agarose beads (Santa Cruz Biotechnology Inc.) were then added and incubated for 2 h at 4 °C. The immunoprecipitates were washed 3 times with buffer A and then homogenized at 4 °C in ice cold TES buffer, and the pIRS1 (1:1000), GR (1:1000) and GRα (1:1000) levels were determined by Western blot analysis as above.

### Statistical analysis

Data (*n* > 6) are expressed as mean ± standard error (SE). Data (*n* ≤ 6) are expressed as mean ± standard deviation (SD). Statistical significance was determined with a two-tailed Student t test, or one-way or two-way ANOVA followed by a Tukey post hoc test. *p* < 0.05 was considered significant.

## Results

### HGSD improves glucose homeostasis in diabetic rats

**Figure 1:**
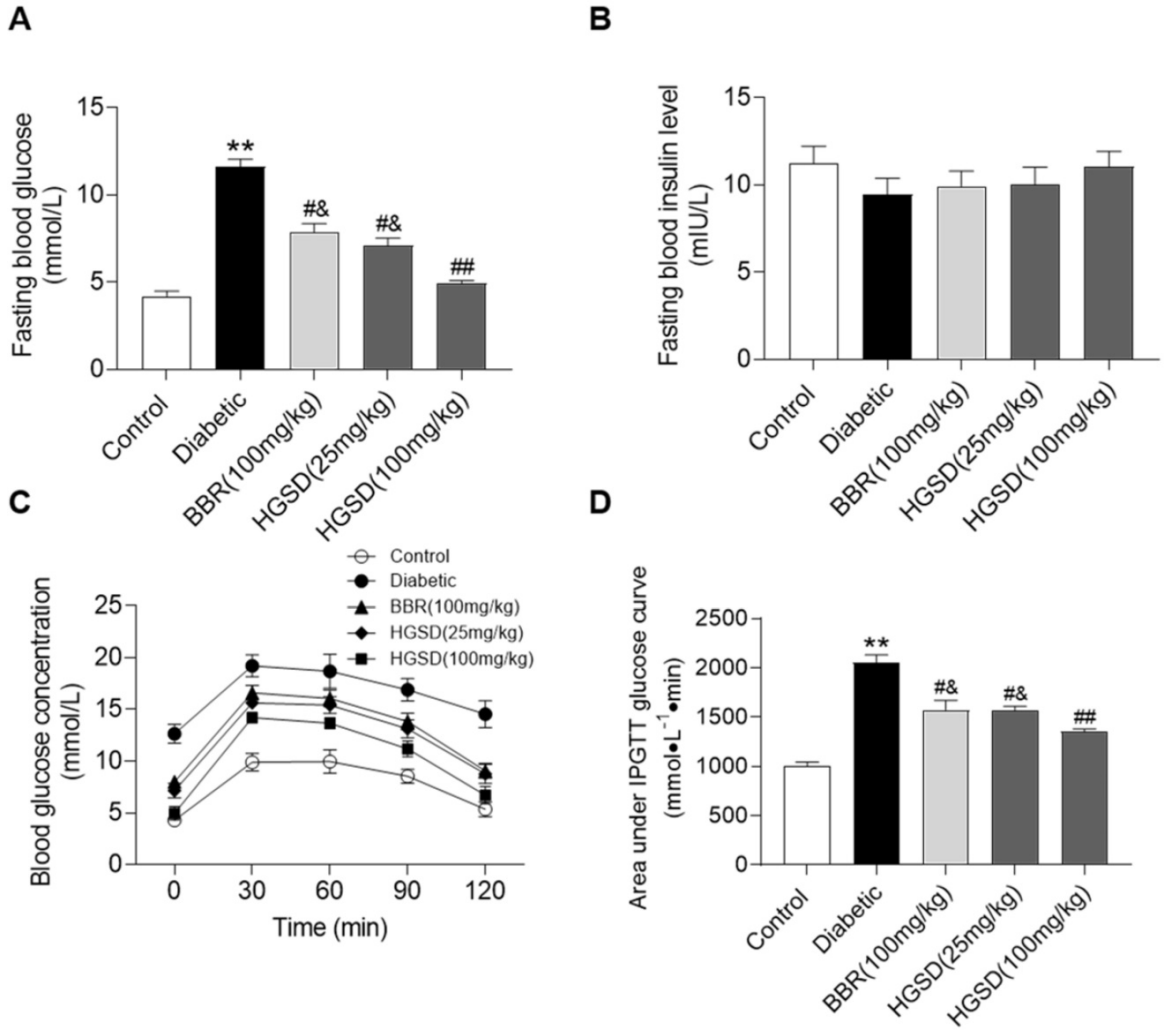
** HGSD is superior to BBR alone in improving glucose homeostasis in diabetic rats.** FBG concentration (**A**), fasting blood insulin level (**B**), blood glucose concentration IPGTT (C) and IPGTT AUC (D) were determined in control and diabetic rats or diabetic rats treated with BBR (BBR 100 mg/kg) or HGSD (25 mg/kg) or HGSD (100 mg/kg). n=8-10. Data are presented as means ± SEM of two or three independent results. *p <0.05, **p <0.01 vs. control; #p <0.05, ##p <0.01 vs. diabetic; &p <0.05 vs. HGSD (100 mg/kg).

We initially examined if BBR or HGSD improved glucose homeostasis in type 2 diabetic rats. Fasting blood glucose (FBG) and insulin levels were determined in control, diabetic and diabetic rats treated for 4 weeks with BBR (100 mg/kg) or HGSD (25 mg/kg or 100 mg/kg). FBG was 2.7-fold higher in diabetic rats compared to control (**Figure 1A**). Treatment of diabetic rats with either BBR (100 mg/kg body weight) or HGSD (25 mg/kg body weight) significantly reduced FBG compared to untreated diabetic animals. Treatment with 100 mg/kg HGSD resulted in a significantly greater reduction in FBG compared to the other treatment groups. Insulin levels were unaltered between all groups (**Figure 1B**). Thus, treatment of type 2 diabetic rats with 100 mg/kg body weight HGSD resulted in a superior hypoglycaemic effect compared to BBR at the equivalent dose.

We next performed an IPGTT in these animals. Rats were injected (i.p.) with glucose and serum glucose levels determined temporally post-injection. Blood glucose concentrations in all animals increased by 30 min after glucose injection and gradually declined over the next 90 min (**Figure 1C**). The diabetic rats exhibited a more marked hyperglycaemia compared to control and drug treated animals. Area under the curve (AUC) for the IPGTT was determined. Diabetic animals exhibited a significantly higher AUC compared to controls (**Figure 1D**). Thus, these animals exhibited insulin resistance. Treatment of diabetic rats with either BBR (100 mg/kg body weight) or HGSD (25 mg/kg body weight) significantly reduced the AUC compared to untreated diabetic animals. Treatment with 100 mg/kg HGSD resulted in a significantly greater reduction in the AUC compared to the other treatment groups. Thus, treatment of diabetic rats with HGSD resulted in a greater attenuation of insulin resistance compared to that of BBR at the equivalent dose.

### HGSD improves insulin signalling in skeletal muscle of diabetic rats

**Figure 2:**
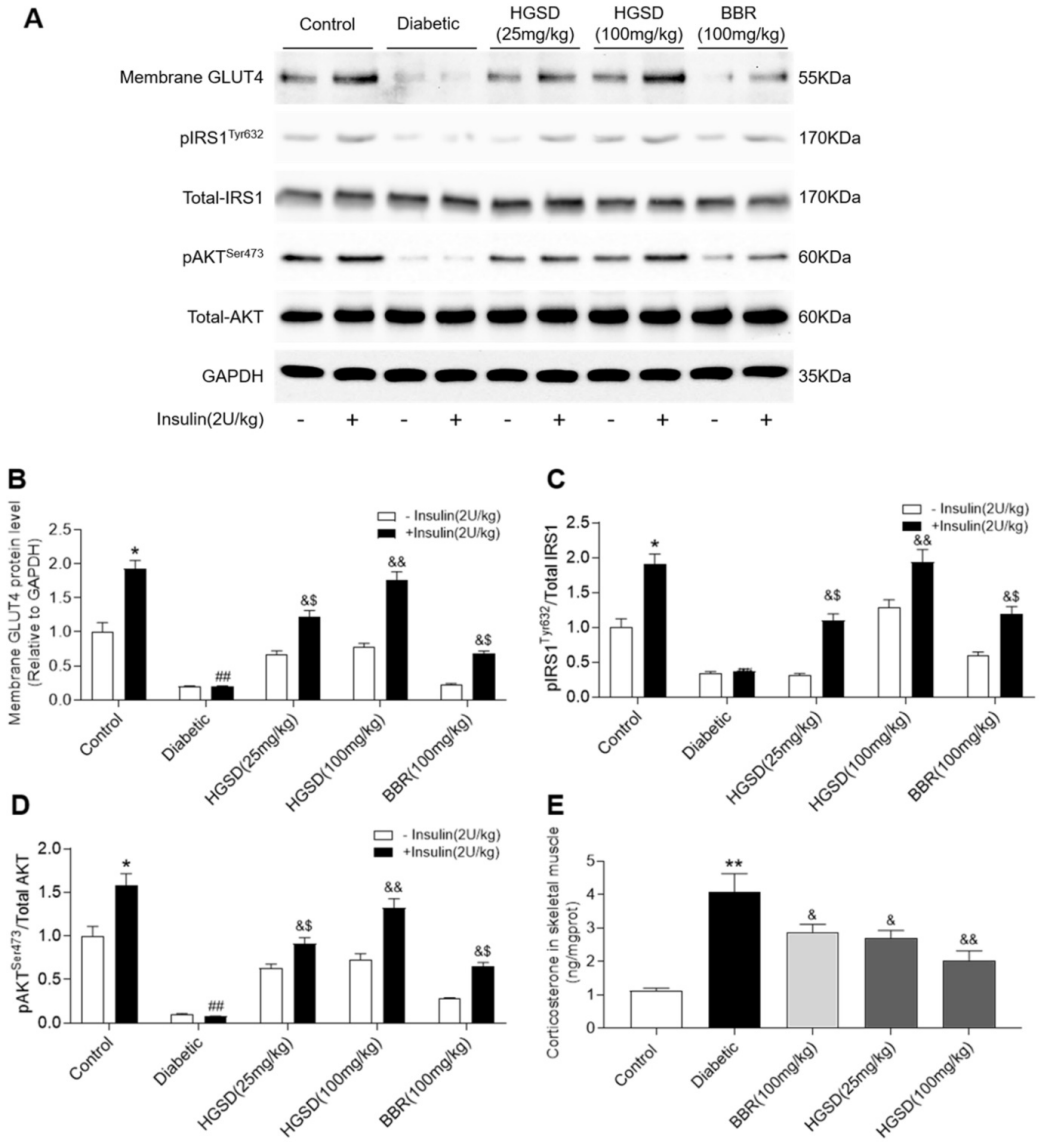
** HGSD is superior to BBR alone in improving impaired insulin-signalling in diabetic rats. A.** Representative Western blot depicting pIRS^Tyr632^, Total-IRS1, pAKT^Ser473^, Total-AKT, Membrane GLUT4 and GAPDH in control and diabetic rats and diabetic rats treated with BBR (BBR 100 mg/kg) or HGSD (25 mg/kg) or HGSD (100 mg/kg)**.** Relative levels of membrane GLUT4 (**B**), pIRS1/IRS1 (**C**), pAKT/AKT (**D**), and skeletal muscle corticosterone (**E**). n=3-6. Data are presented as means ± SD of two or three independent results. *p <0.05, **p <0.01 vs. control minus insulin; #p <0.05, ##p <0.01 vs. control plus insulin; &p <0.05, &&p <0.05 vs. diabetic plus insulin.

To determine how BBR or HGSD improved glucose homeostasis in the above animals, we examined skeletal muscle protein levels of IRS1, pIRS1, AKT, pAKT and membrane GLUT4 before and after insulin stimulation (i.p. injection of insulin 30 min before sacrifice). Membrane GLUT4 was increased by insulin stimulation in the skeletal muscle of control rats (**Figure 2A, B**). Membrane GLUT4 levels were lower in skeletal muscle of diabetic rats and unchanged after insulin stimulation. In contrast, administration of BBR (100 mg/kg) and HGSD (25 mg/kg and 100 mg/kg) increased insulin-stimulated GLUT4 translocation in skeletal muscle compared to diabetic animals. Moreover, treatment of diabetic animals with 100 mg/kg HGSD resulted in a significantly greater insulin-stimulated membrane GLUT4 compared to animals treated with either 25 mg/kg HGSD or 100 mg/kg BBR. Treatment of diabetic rats with 100 mg/kg HGSD appeared to completely restore the level of insulin-stimulated membrane GLUT4 to that of control levels. Thus, HGSD better improved insulin signalling compared to that of BBR at the equivalent dose.

Total AKT and IRS1 protein levels were unaltered between all groups (**Figure 2A**). In contrast, phosphorylation of AKT and IRS1 was increased in skeletal muscle of control but not diabetic rats after insulin stimulation. Treatment of diabetic animals with BBR or HGSD increased insulin-stimulated phosphorylation of AKT. Moreover, treatment of diabetic animals with 100 mg/kg HGSD resulted in a significantly greater insulin-stimulated AKT phosphorylation than compared to animals treated with either 25 mg/kg HGSD or 100 mg/kg BBR. Treatment of diabetic rats with 100 mg/kg HGSD completely restored the insulin-stimulated ratio of pIRS1/IRS1 and pAKT/AKT to that of controls (**Figure 2C, D**).

Excess GC may play an important role in the pathogenesis of obesity and type 2 diabetes by directly antagonizing insulin action and inhibiting insulin release. Increased circulating cortisol levels may impair insulin sensitivity and promote differentiation of adipocyte precursor cells into fat cells 27, 28. In addition, increased GC action on skeletal muscle has been shown to impair insulin-signalling through a variety of pathways 29, 30. We thus examined the corticosterone levels in skeletal muscle of the above animals. Skeletal muscle corticosterone levels were significantly increased in diabetic rats compared to controls (**Figure 2E**). Administration of BBR (100 mg/kg) and HGSD (25 mg/kg and 100 mg/kg) reduced corticosterone levels in skeletal muscle compared to diabetic animals. Moreover, treatment of diabetic animals with 100 mg/kg HGSD resulted in a significantly greater reduction in skeletal muscle corticosterone levels compared to animals treated with either 25 mg/kg HGSD or 100 mg/kg BBR. These results indicated that the anti-diabetic effects of HGSD may be related to its ability to attenuate GC-mediated insulin-resistance.

### HGSD increases glucose consumption and uptake in insulin-resistant C2C12 cells

**Figure 3:**
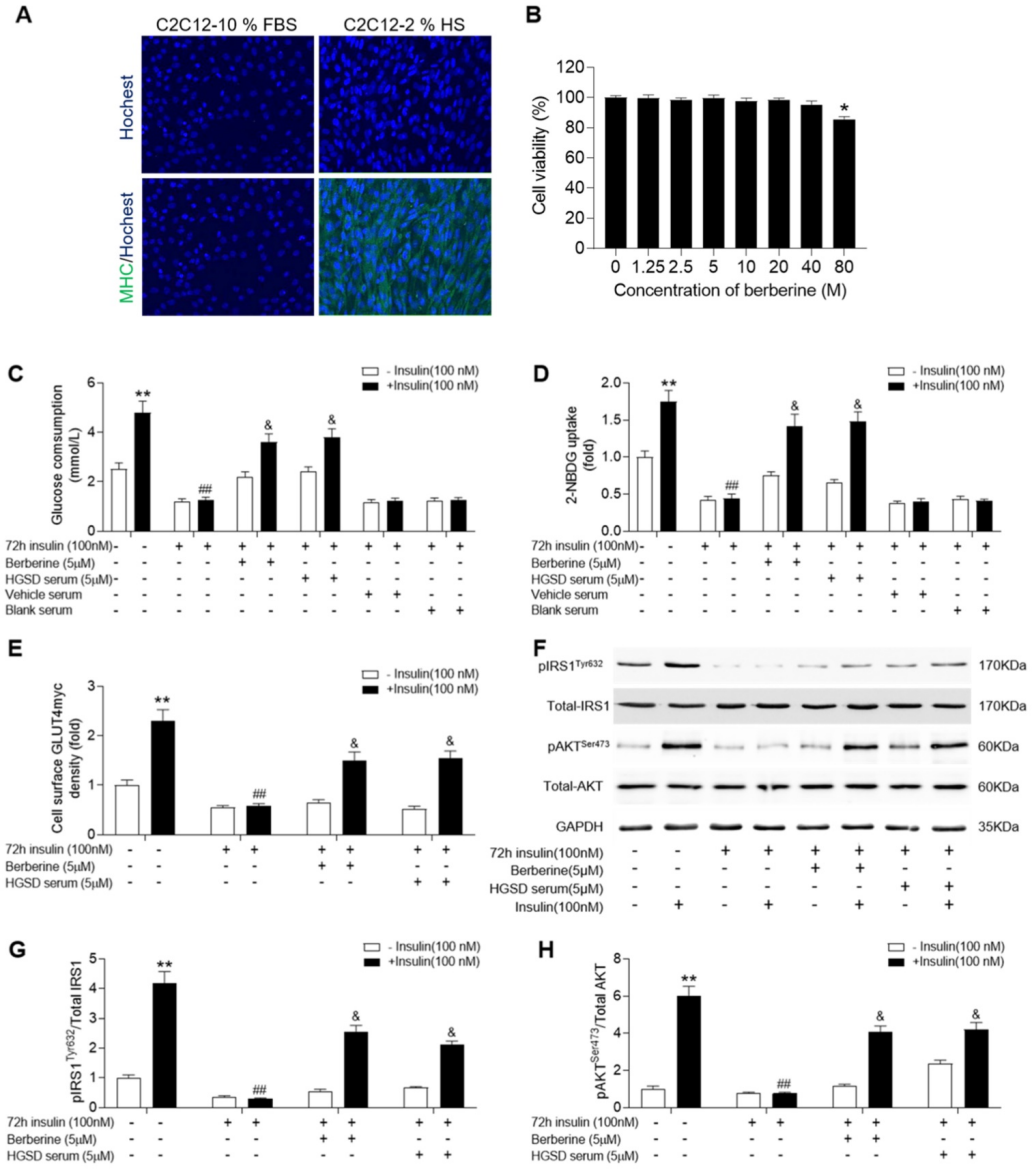
** HGSD increases glucose consumption and improves insulin signalling in insulin-resistant C2C12 cells. A.** Hochest staining for nuclei and myosin heavy chain (MHC) in C2C12 cells cultured in medium supplemented with either 10% FBS or 2% HS. **B.** Percent viability of C2C12 cells after differentiation incubated with various concentrations (0-80 µM) of BBR for 24 h. C2C12 cells were incubated in the absence (-) or presence (+) of insulin (100 nM) for 72 h then incubated plus or minus BBR or HGSD conditioned serum or vehicle serum or blank serum (n=6). Glucose consumption (**C**; n=6), glucose uptake (**D**; n=6), membrane GLUT4 levels (**E**) and IRS1, pIRS1, AKT, pAKT and GAPDH levels (**F**), ratio of pIRS1/IRS1 (**G**) and ratio of pAKT/AKT (**H**) determined in the absence (open bars) or presence (closed bars) of 100 nM insulin stimulation for 30 min (n=3). Data are presented as means ± SD of two or three independent results. *p <0.05, **p <0.01 vs. control without insulin stimulation; #p <0.05, ##p <0.01 vs. control group with insulin stimulation; &p <0.05 vs. insulin stimulation.

C2C12 cells exhibit a similar insulin signalling pathway to that of neonatal myotubes and high levels of insulin exposure result in insulin resistance in C2C12 cells. We examined if BBR or HGSD improved glucose homeostasis in insulin-resistant C2C12 cells. C2C12 cells grown in medium supplemented with 10% FBS (growth-promoting medium) exhibited a myoblast phenotype and remained mononucleated (**Figure 3A**). Differentiation of C2C12 cells in medium supplemented with 2% horse serum (HS) resulted in increased fusion and more elongated cells with multiple nuclei. To further characterize differentiation induction expression of myosin heavy chain (MHC) was examined. Differentiated C2C12 cells exhibited an increased expression of MHC. Thus, the C2C12 cells used in this study were differentiated into myotubes. The cytotoxicity of BBR was examined in differentiated C2C12 cells. BBR was non-cytotoxic at up to 40 µM (**Figure 3B**). In addition, no abnormal morphological features were observed in cells treated with 1.25-40 μM BBR as assessed by light microscopy analysis (data not shown). Based on the above results and our previous studies of BBR on promoting glucose consumption and uptake of C2C12 myotubes, a 5 μM concentration of BBR was chosen for all subsequent experiments.

C2C12 myotubes were incubated in the absence or presence of 100 nM insulin for 72 h to induce insulin-resistance and then glucose consumption and glucose uptake examined in cells incubated in the absence or presence of BBR or HGSD conditioned serum. Both glucose consumption and uptake were increased by insulin treatment in insulin-sensitive control C2C12 cells (**Figure 3C, D**). In contrast, cells pre-incubated with insulin for 72 h were unresponsive to insulin-stimulated glucose consumption and uptake. Incubation of insulin-resistant cells with either 5 uM BRR or HGSD conditioned serum increased insulin-stimulated glucose consumption and uptake. Cell incubated with vehicle serum (prepared from animals gavaged with saline alone) or blank serum (prepared from untreated animals) remained unresponsive to insulin-stimulated glucose consumption and uptake. These results indicate that HGSD improves insulin-stimulated glucose consumption and uptake in insulin resistant C2C12 cells.

### HGSD improves insulin signalling in insulin-resistant C2C12 cells

To determine how HGSD improved glucose consumption and uptake in insulin-resistant C2C12 cells, we examined protein levels of IRS1, pIRS1, AKT, pAKT and cell surface GLUT4 before and after insulin stimulation in the above cells. GLUT4 on the cell surface of insulin-sensitive C2C12 cells increased significantly after insulin (100 nM) stimulation compared to controls (**Figure 3E**). Incubation of insulin-resistant cells with either BBR or HSGD conditioned serum increased insulin-stimulated GLUT4 density on the cell surface compared to untreated cells. Total AKT and IRS1 protein levels were unaltered between all groups (**Figure 3F**). In contrast, phosphorylation of AKT and IRS1 was increased in control C2C12 cells but not in insulin resistant cells. Treatment of insulin-resistant C2C12 cells with HGSD conditioned serum increased insulin-stimulated phosphorylation of AKT and IRS1 compared to untreated cells, which was in line with berberine treatment group (**Figure 3G, H**).

**Figure 4:**
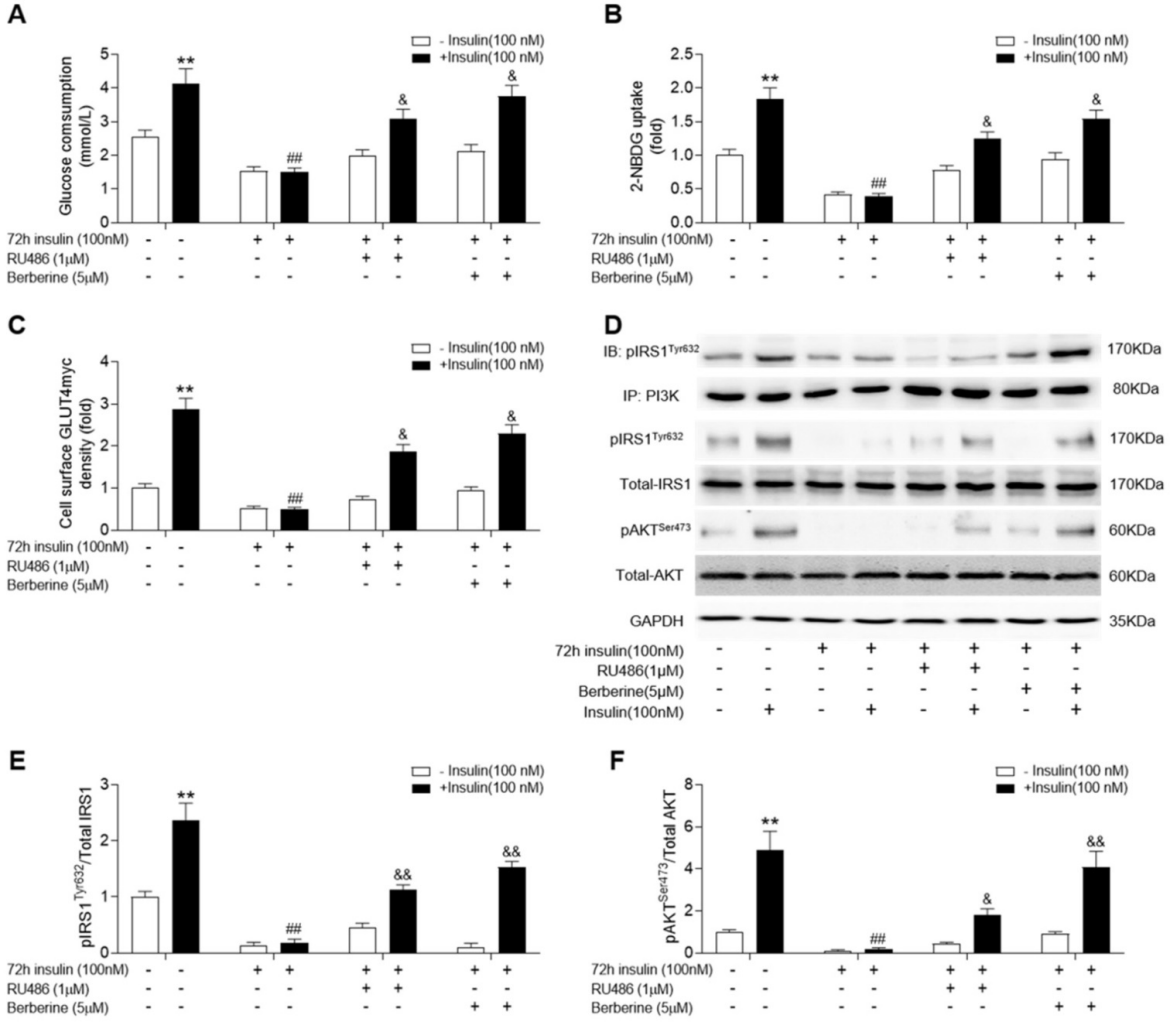
**Berberine improves insulin signalling in insulin-resistant C2C12 cells similar to RU486.** C2C12 cells were incubated in the absence (-) or presence (+) of insulin (100 nM) for 72 h then incubated plus or minus BBR (active ingredient of HGSD) or RU486. Glucose consumption (**A**; n=6), glucose uptake (**B**; n=6), membrane GLUT4 levels (**C**) and IRS1, pIRS1, AKT, pAKT and GAPDH levels (**D**) in control and insulin-resistant C2C12 cells were determined in the absence (open bars) or presence (closed bars) of insulin stimulation (100 nM) for 30 min (n=3). Ratio of pIRS1/IRS1 (C) and ratio of pAKT/AKT (D) in control and insulin-resistant C2C12 cells incubated in the absence (open bars) or presence (closed bars) of insulin stimulation (100 nM) for 30 min. Data are presented as means ± SD of two or three independent results. *p <0.05, **p <0.01 vs. control without insulin stimulation; ##p <0.01 vs. control with insulin stimulation; &p <0.05 vs insulin stimulation.

Given that corticosterone levels were significantly increased in skeletal muscle of diabetic rats, we used RU486, an antagonist of GR, to treat insulin-resistant C2C12 cells. In addition, we examined whether blockade of GR action could improve insulin signalling and if BBR (the active ingredient of HGSD) had a similar effect to that of RU486. Both BBR and RU486 increased glucose consumption and uptake in insulin-resistant C2C12 cells induced by 72 h incubation of 100 nM insulin in basal and insulin stimulated state (**Figure 4A, B**). While treatment of insulin-resistant C2C12 cells with BBR improved insulin-stimulated the association of pIRS1 and PI3K, GLUT4 translocation, ratio of pIRS1/IRS1 and pAKT/AKT remained similar to that of insulin-resistant cells treated with RU486 (**Figure 4C-F**). Thus, HGSD improves insulin signalling in insulin-resistant C2C12 cells and this may be related to its regulation of GR action.

### HGSD improves glucose homeostasis in DEX-treated mice

**Figure 5:**
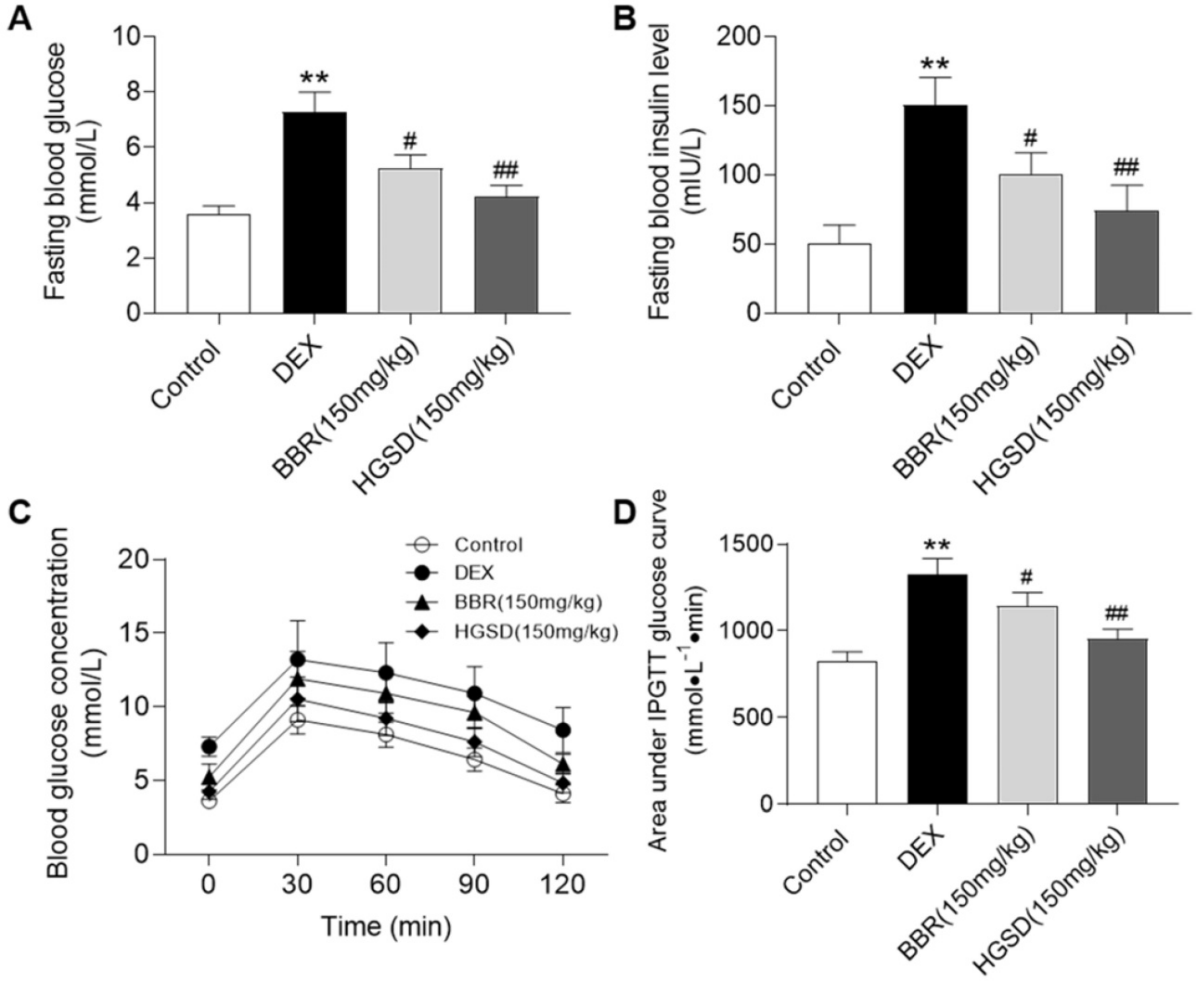
** HGSD improves glucose homeostasis in DEX-treated mice.** FBG concentration (**A**), fasting serum insulin level (**B**), blood glucose concentration OGTT (**C**) and OGTT AUC (**D**) were determined in control and DEX-treated mice or DEX-treated mice fed BBR (BBR 150 mg/kg) or HGSD (150 mg/kg). n=8-10. Data are presented as means ± SEM of two or three independent results. *p <0.05, **p <0.01 vs. control; #p <0.05, ##p <0.01 vs. DEX-treated.

Dexamethasone administration is well known to induce insulin resistance by activating gluconeogenesis 31, 32. To further examine the promoting effects of HGSD on glucose uptake in skeletal muscle, we treated dexamethasone-induced insulin resistance mice with HGSD. Mice were made diabetic by treatment with DEX and treated with BBR or HGSD and FBG and fasting serum insulin levels determined. FBG levels were elevated in DEX-treated mice compared to control (**Figure 5A**). Treatment of DEX-treated mice with either BBR (150 mg/kg body weight) or HGSD (150 mg/kg body weight) significantly reduced FBG compared to DEX-treated animals. Treatment with HGSD resulted in a significantly greater reduction in FBG compared to BBR. Fasting insulin levels were elevated in DEX-treated mice compared to control (**Figure 5B**). Treatment of DEX-treated mice with either BBR (150 mg/kg body weight) or HGSD (150 mg/kg body weight) significantly reduced fasting insulin levels compared to DEX-treated animals. Treatment with HGSD resulted in a significantly greater reduction in fasting insulin levels compared to BBR.

We next performed an OGTT in these animals. Mice were gavaged with glucose and serum glucose levels determined temporally post-gavage. Blood glucose concentrations in all animals increased by 30 min after glucose administration and gradually declined over the next 90 min (**Figure 5C**). The DEX-treated mice exhibited a more marked hyperglycaemia compared to control and drug treated animals. Area under the curve (AUC) for the OGTT was determined. DEX-treated mice exhibited a significantly higher AUC compared to controls (**Figure 5D**). Thus, these animals exhibited insulin resistance. Treatment of DEX-treated mice with either BBR or HGSD significantly reduced the AUC compared to DEX-treated mice. Treatment with HGSD resulted in a significantly greater reduction in the AUC compared to BBR. Thus, treatment of diabetic mice with HGSD resulted in a superior hypoglycaemic effect compared to BBR at the equivalent dose.

### HGSD improves insulin signalling in DEX-treated mice

**Figure 6:**
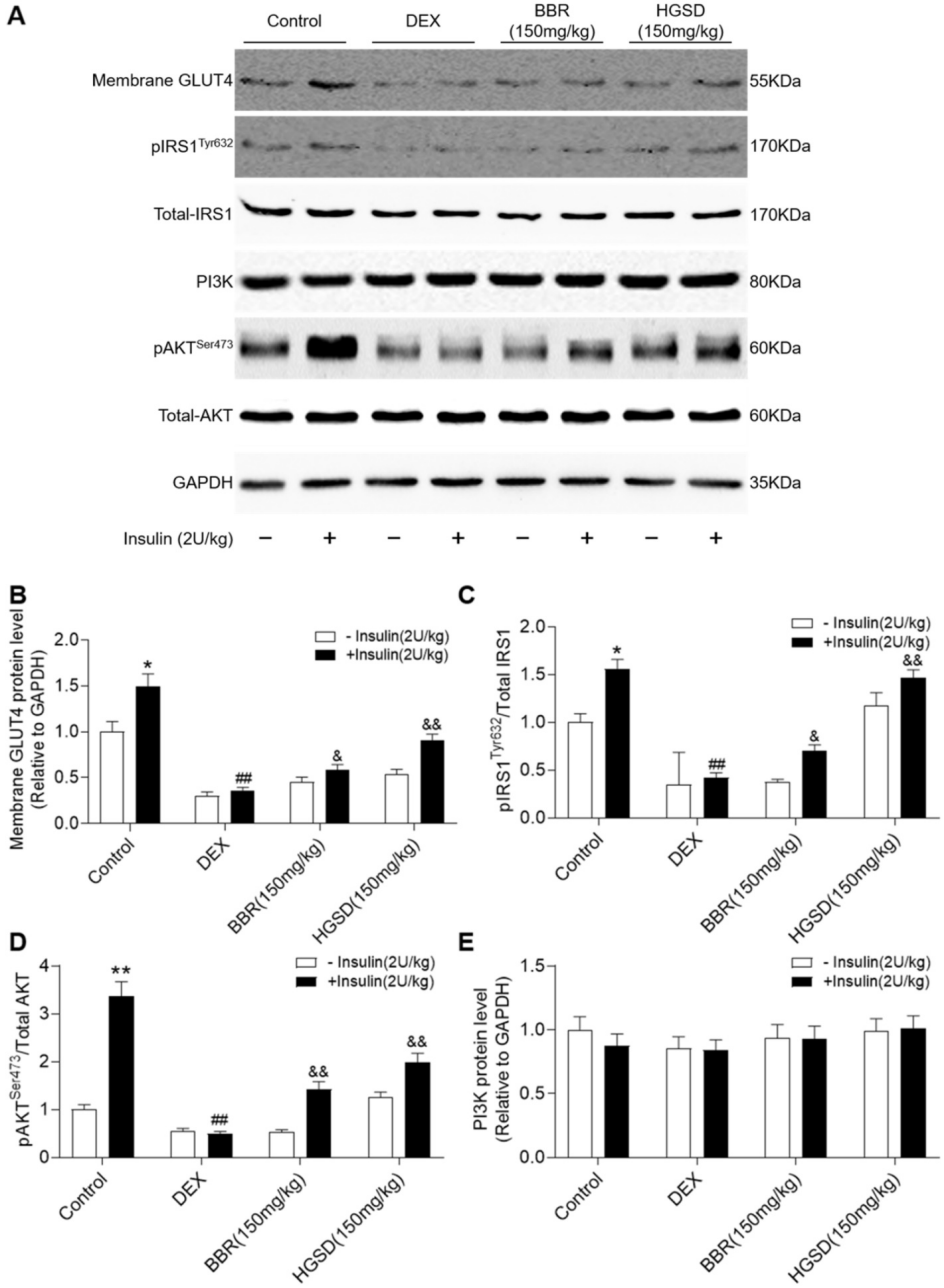
** HGSD improves skeletal muscle insulin signalling in DEX-treated mice. A.** Skeletal muscle membrane GLUT4, IRS1, pIRS1, AKT, pAKT, PI3K, and GAPDH levels in Control (Control) or DEX-treated mice (DEX) or DEX-treated mice treated with BBR (BBR) or DEX-treated mice treated with HGSD (HGSD) incubated in the absence (-) or presence (+) of insulin stimulation. Control (open bars) and insulin-stimulated (closed bars) levels of skeletal muscle membrane GLUT4 (**B**) ratio of pIRS1/IRS1 (**C**), ratios of pAKT/AKT and PI3K (**D**) in the above animals. n=3. Data are presented as means ± SD of two or three independent results. *p <0.05, **p <0.01 vs. control group without insulin stimulation; #p <0.05, ##p <0.01 vs. control with insulin stimulation; &p <0.05, &&p <0.01 vs. DEX-treated group with insulin stimulation.

To determine how HGSD improved glucose consumption and uptake in DEX-treated mice, we examined protein levels of IRS1, pIRS1, AKT, pAKT, PI3K and membrane GLUT4 before and after insulin stimulation in skeletal muscle. Membrane GLUT4 increased significantly after insulin stimulation in control animals (**Figure 6A, B**). In contrast, membrane GLUT4 was significantly decreased in DEX-treated and insulin-stimulated DEX-treated mice compared to controls. Membrane GLUT4 levels were increased in insulin-stimulated DEX-treated mice incubated with either BRR or HGSD compared to untreated DEX-treated mice. Total AKT and IRS1 protein levels were unaltered between all groups (**Figure 6A**). In contrast, phosphorylation of AKT and IRS1 was increased by insulin stimulation in skeletal muscle of control mice but not in DEX-treated mice. Treatment of DEX-treated mice with BBR or HGSD increased insulin-stimulated phosphorylation of AKT and IRS1. Treatment of DEX-treated mice with BBR or HGSD increased insulin-stimulated ratio of pIRS1/IRS1 and pAKT/AKT (**Figure 6C, D**). Treatment of DEX-treated mice with HGSD appeared to result in a greater increase in the ratio of pIRS1/IRS1 and pAKT/AKT compared to DEX-treated mice treated with BBR at the equivalent dose. Total PI3K protein levels were unaltered between all groups (**Figure 6E**). Thus, HGSD treatment improves insulin signalling in insulin-resistant DEX-treated mice.

### HGSD attenuates association of PI3K with GR and GRα in skeletal muscle of DEX-treated mice

**Figure 7:**
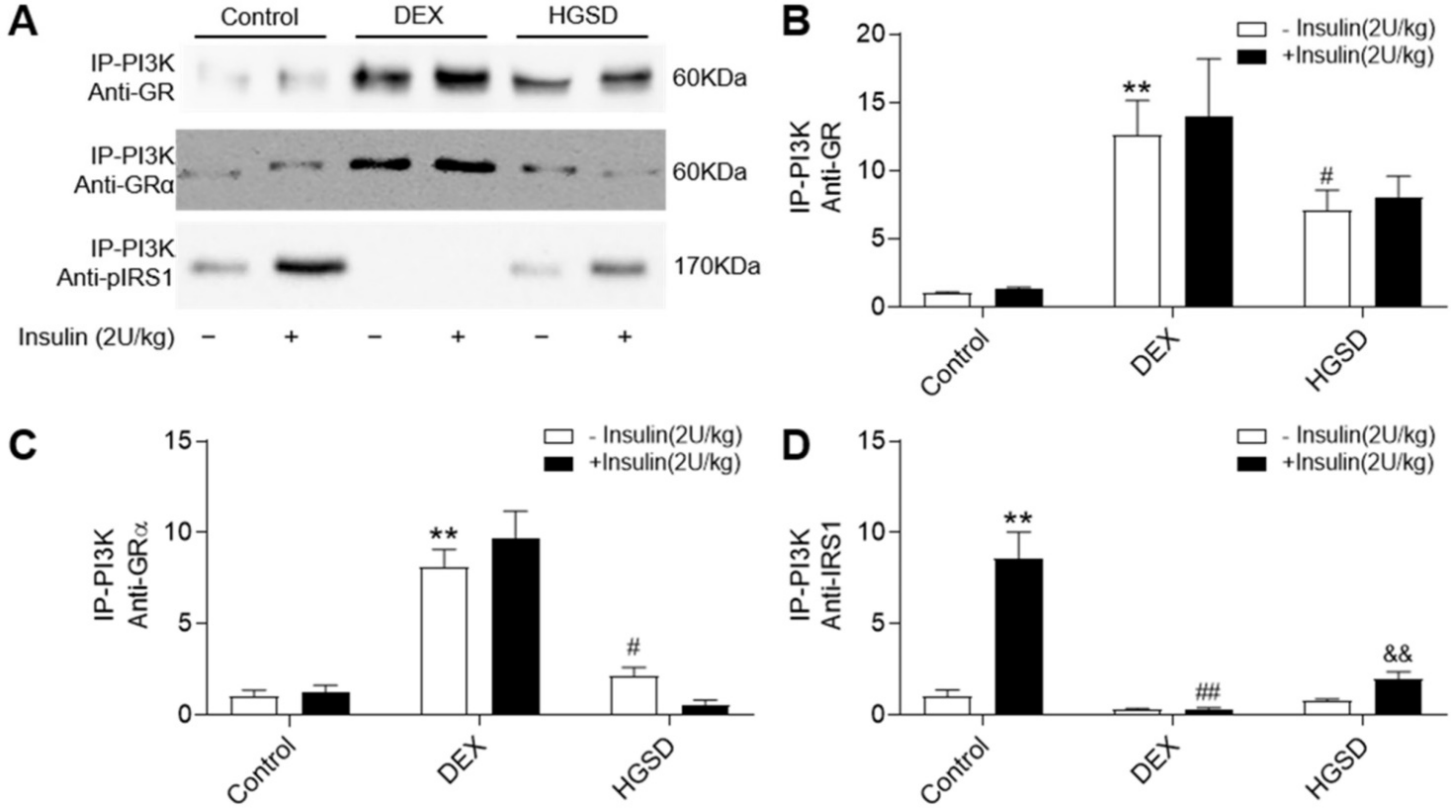
** HGSD attenuates association of PI3K with GR and GRα in skeletal muscle of DEX-treated mice. A.** Skeletal muscle PI3K-associated GR, GRα and pIRS1 in control (Control) or DEX-treated (DEX) mice or DEX-treated mice treated with HGSD (HGSD) stimulated in the absence (-) or presence (+) of insulin stimulation. **B.** PI3K-GR association. **C.** PI3K-GRα association. **D.** PI3K-pIRS1 association. n=3. Data are presented as means ± SD of two or three independent results. *p <0.05, **p <0.01 vs. control without insulin stimulation; #p <0.05, ##p <0.01 vs. control with insulin stimulation; &p <0.05 vs. DEX with insulin stimulation.

Since HGSD treatment improved glucose homeostatsis and insulin signalling in skeletal muscle of insulin-resistant DEX-treated mice, we examined if this was mediated through regulation of GR and PI3K association, cytoplasmic pIRS/PI3K association and GR/PI3K association. Insulin stimulation promoted an increase in association of PI3K with IRS1 in skeletal muscle and this was attenuated in DEX-treated mice (**Figure 7A, D**). Treatment of DEX-treated mice with HGSD increased insulin-stimulated association of PI3K with IRS1. PI3K-associated GR and GRα was increased and PI3K-associated pIRS1 decreased in skeletal muscle of DEX-treated mice compared to control (**Figure 7A-C**). Treatment of DEX-treated mice with HGSD reduced the association of PI3K with GR or GRα. Thus, HGSD may improve insulin signalling in insulin-resistant DEX-treated mice, in part, through attenuating the association of PI3K with GR and GRα.

## Discussion

Excess GC in skeletal muscle may play an important role in the development of Type 2 diabetes. However, the molecular mechanism for the development of insulin resistance mediated through GC-mediated GR action is not fully understood. Moreover, it was unknown whether increased bioavailable preparations of BBR, such as HGSD, could attenuate insulin resistance via restoring GC-mediated GR function in skeletal muscle. In the current study, we demonstrated that elevated GC in skeletal muscle resulted in insulin resistance via regulating non-genomic effects of GR and that HGSD attenuated this and improved insulin resistance. The principal findings of our study are that (1) DEX-mediated GR and GRα association with PI3K inhibits the association of PI3K with pIRS1 in skeletal muscle and this may contribute to development of insulin resistance, and (2) Treatment with HGSD improves insulin resistance through decreased association of GR and GRα with PI3K and subsequently increased association of PI3K with IRS1.

Skeletal muscle is one of the key tissues that mediates glucose uptake and consumption and largely regulates glucose homeostasis 33. Therefore, excess GC-mediated GR action in skeletal muscle may affect its insulin sensitivity. It was previously reported that expression and action of GRα in human skeletal muscle was closely correlated with various indexes of the metabolic syndrome. These authors reported a negative correlation of glucose consumption rate and positive correlation of the BMI and body fat with GRα level 30. These findings substantiate the importance of GR in the pathogenesis of insulin resistance and obesity and thereby implicate the GR as a potential target for the treatment of Type 2 diabetes and obesity. In addition, it was observed that the association of GR with PI3K resulted in an additional suppression of IRS-1 associated PI3K and Akt activities leading to muscle atrophy 34. However, how GR or GRα results in insulin resistance via affecting the insulin signalling pathway is poorly understood. In the present study, we evaluated GC-mediated GR action and the insulin signalling pathway in two *in vivo* and one *in vitro* model of insulin resistance or diabetes.

In high-fat diet STZ-treated diabetic rats, the insulin signalling pathway was impaired and this was associated with elevated levels of skeletal muscle GC. Treatment of these animals with HGSD attenuated insulin-resistance and improved insulin signalling. In insulin-resistant C2C12 cells, both HGSD and RU486 attenuated insulin-resistance to a similar extent. Similar results to previous study, we found a promoting effect of berberine on basal glucose uptake in insulin-resistant C2C12 cells 35. DEX decreased insulin-stimulated glucose uptake by reducing the levels of the tyrosine-phosphorylated IR and total IRS-1 proteins in murine skeletal muscle 36. The activities of phosphoinositide-3-kinase (PI3K) and AKT, two key signalling molecules downstream of IR and IRS-1, were also decreased 36-38. Moreover, berberine could reduce insulin resistance-induced by DEX by methylated state of HIF3A and mitochondrial function 14, 39. And It was previously reported that the anti-inflammation activity of berberine was not based on the modulation of DEX binding to GR 40, 41. In present study, we observed that insulin-mediated phosphorylation of IRS1 was attenuated in skeletal muscle of insulin-resistant DEX-treated mice implying an impaired insulin signalling pathway. Furthermore, we showed that the DEX-mediated insulin resistance may be due, in part, to an increase in association of GR/GRα with PI3K and a decrease in association of PI3K with pIRS1 and that HGSD attenuated this effect. These data support a direct action of the GR on the insulin signalling pathway, which is in the regulation of HGSD by disassociation GR/GRα with PI3K.

We previously showed that treatment of diabetic animals with BBR reduced the mRNA and protein levels of PEPCK and the hepatic glucose output via decreasing its transcription mediated by the GR in diabetic rats. The results suggested that BBR may potentially serve as a therapeutic drug for diabetes targeting the GR. However, the low oral bioavailability of BBR and its unclear anti-diabetic mechanism limits its clinical application 16. We subsequently developed a new drug delivery system and confirmed that the bioavailability of BBR could be significantly enhanced using HGSD preparations 15. In the present study, it was observed that HGSD exhibited a more enhanced hypoglycaemic effect than an equivalent dose of commercial BBR, effectively one quarter the dose of that of BBR. In diabetic rats, HGSD ameliorated insulin resistance by increasing GLUT4 translocation and phosphorylation of IRS1 and AKT, while decreasing GC levels in skeletal muscle. In insulin-resistant C2C12 cells, HGSD increased glucose uptake and consumption and improved insulin resistance. Finally, in our DEX-treated mouse model of insulin resistance HGSD markedly improved insulin resistance by decreasing the association of GR/GRα with PI3K and increasing the association of pIRS1 with PI3K.

The mechanism for GR-related insulin resistance in skeletal muscle remains largely unknown and there are limited anti-diabetic drug candidates targeting the GR. In the present study, we identify a new mechanism of insulin resistance mediated by excess GC-activated GR in the skeletal muscle of diabetic rats and mice. The increase in the association of GR/GRα with PI3K and decrease in association of pIRS1 with PI3K may play vital roles in the development of insulin resistance in skeletal muscle. HGSD, a new drug delivery system of BBR with its enhanced bioavailability reduced PI3K-associated GR and may be a promising candidate drug for the treatment of Type 2 diabetes.

## Highlights

- GR and GRα could co-precipitated with PI3K, which inhibits the association of PI3K with pIRS1 in skeletal muscle;
- The competitive inhibition of GR and GRα for the association of PI3K with pIRS1 blocks insulin-dependent glucose consumption pathway in skeletal muscle, which contribute to development of insulin resistance;
- Treatment with HGSD improves insulin resistance through decreased association of GR and GRα with PI3K, and subsequently increased glucose consumption in skeletal muscle.

## Abbreviations

BBR: Berberine
DEX: dexamethasone
HFD: high-fat diet
HGSD: Huang-Gui solid dispersion
HS: horse serum
GC: Excess glucocorticoid
MHC: myosin heavy chain
SC: sodium caprate
